# Bronchodilator response assessment through impulse oscillometry system and spirometry in children and adolescents with cystic fibrosis

**DOI:** 10.1590/1984-0462/2024/42/2023162

**Published:** 2024-05-27

**Authors:** Tayná Castilho, José Dirceu Ribeiro, Renata Maba Gonçalves Wamosy, Juliana Cardoso, Gabriela Castilhos Ducati, Camila Isabel Santos Schivinski

**Affiliations:** a Universidade Estadual de Campinas, Campinas, SP, Brazil.; b Universidade do Estado de Santa Catarina, Florianópolis, SC, Brazil.

**Keywords:** Cystic fibrosis, Bronchodilator agents, Respiratory mechanics, Pulmonary function tests, Fibrose cística, Agentes broncodilatadores, Mecânica respiratória, Testes de função pulmonar

## Abstract

**Objective:**

To investigate the effect of bronchodilator on the respiratory mechanics and pulmonary function of children and adolescents with cystic fibrosis.

**Methods:**

Cross-sectional study on clinically stable children and adolescents with cystic fibrosis aged from six to 15 years. Participants underwent impulse oscillometry and spirometry evaluations before and 15 minutes after bronchodilator inhalation. The Kolmogorov-Smirnov test was applied to verify the sample distribution, and the Student's t-test and Wilcoxon test were used to compare the data before and after bronchodilator inhalation.

**Results:**

The study included 54 individuals with a mean age of 9.7±2.8 years. The analysis showed a statistically significant improvement in impulse oscillometry and spirometry parameters after bronchodilator inhalation. However, according to the American Thoracic Society (ATS) and European Respiratory Society (ERS) recommendations (2020 and 2021), this improvement was not sufficient to classify it as a bronchodilator response.

**Conclusions:**

The use of bronchodilator medication improved respiratory mechanics and pulmonary function parameters of children and adolescents with cystic fibrosis; however, most patients did not show bronchodilator response according to ATS/ERS recommendations.

## RESUMO

**Objetivo:**

Investigar o efeito do broncodilatador na mecânica respiratória e função pulmonar de crianças e adolescentes com fibrose cística.

**Métodos:**

Estudo transversal em crianças e adolescentes com fibrose cística clinicamente estáveis, com idade entre seis e 15 anos. Os participantes realizaram avaliações por meio do sistema de oscilometria de impulso e espirometria antes e 15 minutos depois da inalação de broncodilatador. Foi aplicado o teste Kolmogorov-Smirnov para verificar a distribuição da amostra. Para comparar os dados antes e depois do broncodilatador foram utilizados os testes *t* de Student e Wilcoxon.

**Resultados:**

Participaram do estudo 54 indivíduos com média de idade de 9,7±2,8 anos. Houve melhora estatisticamente significativa nos parâmetros do oscilometria de impulso e espirometria após a inalação com broncodilatador. No entanto, de acordo com recomendações da American Thoracic Society (ATS) e European Respiratory Society (ERS) (2020 and 2021), essa melhora não foi suficiente para classificar como reposta broncodilatadora.

**Conclusões:**

O uso de medicamento broncodilatador melhorou a mecânica respiratória e função pulmonar de crianças e adolescentes com fibrose cística, no entanto a maioria da amostra não apresentou resposta ao broncodilatador de acordo com as recomendações da ATS/ERS.

## INTRODUCTION

Bronchodilators (BDs) are commonly used in treating respiratory diseases to relax airway smooth muscles and alleviate symptoms of bronchoconstriction, such as dyspnea.^1,2^ The use of BDs in diseases like asthma and chronic obstructive pulmonary disease (COPD) is well-established in the scientific literature.^1,3^ However, the effectiveness of BDs in people with cystic fibrosis (pwCF) remains a topic of debate, despite being frequently prescribed in clinical practice.^4,5^

Barry and Flume (2017) raised the following arguments for the prescription of BD to individuals with cystic fibrosis (CF): the presence of asthma associated with CF; the obstructive pulmonary characteristic of CF; the bronchoconstriction caused by inhalation of medications such as antibiotics and hypertonic saline solution; and the mucociliary clearance effect.^5^ Despite these theoretical issues, the authors affirmed that the evidence for BD use in CF must be better explored.^5^

Two systematic reviews were conducted concerning BD use in pwCF.^4,6^ One focused on the effect of long-acting inhaled BDs and analyzed four clinical trials.^4^ The authors concluded that there was no improvement in the forced expiratory volume in one second (FEV_1_) and the quality of the evaluated papers was poor.^4^ The other systematic review investigated short-acting inhaled BDs and analyzed 11 clinical trials.^6^ Although the trials showed an improvement in FEV_1_, it was not reported if such improvement achieved the threshold for a bronchodilator response (BDR) according to the American Thoracic Society and European Respiratory Society (ATS/ERS)^7^. Both systematic reviews informed that there was little evidence on the BD effect in CF. Nevertheless, none of the studies included in those systematic reviews evaluated BD effects on respiratory mechanics.

One way of measuring the BD effect on respiratory mechanics is via the impulse ocillometry system (IOS), a tool that assesses the airway's resistance and reactance during tidal breathing.^8^ The ERS recently released a document that establishes cut-off points to identify if changes in respiratory mechanics are a consequence of BD inhalation.^9^ Since IOS does not require forced expiration, it can be used in conjunction with spirometry to detect a BDR in pwCF.^10^

This study aimed to investigate the BDs effect on the spirometry and IOS parameters of children and adolescents with CF. As the BDs’ mechanism of action is to relax the airway smooth muscles, we hypothesized that there would be an improvement in airway resistance after BD inhalation.

## METHOD

This is a cross-sectional study performed in a CF referral center in Brazil, from June 2017 to November 2018. It was approved by the referral center's Ethics Committee (CAAE: 80800217.4.0000.5361), and written informed consent was obtained from the patients and their guardians.

This study included individuals aged six to 15 years with a confirmed CF diagnosis^12^, and the convenience sampling method was used. Individuals were excluded from the sample if they could not use BD at the moment of evaluation or if they presented an acute pulmonary exacerbation as identified by the Cystic Fibrosis Clinical Score (CFCS)^12^ and the Cystic Fibrosis Foundation Score (CFFS).^13^

The following data were obtained from the medical records: pathogen colonization (of at least one pathogen); genetic variant (with the presence of at least one allele with F508del variant); and disease severity (categorized as "excellent", "good", "average", "poor", or "severe" according to the Schwachman-Doerschuk Score [SDS]).^14^ The use of BDs and/or steroid medications commonly prescribed to treat asthma was also recorded. Anthropometric measurements of weight, height, and body mass index (BMI) were collected. The BMI was presented in kg/m^2^ and z-scores (the latter, according to World Health Organization).^15^ Participants were categorized as "eutrophic", "overweight", "obesity", "thinness", or "severe thinness".^15^

Respiratory mechanics and pulmonary function were evaluated by IOS and spirometry, respectively. Following the ATS/ERS recommendations,^7,9^ these evaluations were performed on a pneumatograph Master Screen IOS (Erich Jaeger, Würtzburg, Germany^®^). In order to avoid interference of forced maneuvers on the oscillometric parameters, IOS evaluation was always performed before spirometry. The oscillometric parameters were impedance at 5Hz (Z5), resistance at 5Hz (R5), resistance at 20Hz (R20), and reactance at 5Hz (X5). The R5 and R20 parameters are commonly referred to as total airway resistance and central airway resistance, respectively. The reference IOS parameters were computed via Assumpção et al. reference equation^16^ and each parameter was presented in absolute and predicted percentage (pred%).

Regarding spirometry, the following parameters were recorded: forced vital capacity (FVC), FEV_1_, and forced expiratory flow at 25 to 75% of FVC (FEF_25-75_). The reference values were obtained through the Global Lung Function Initiative (GLI)^17^, and the parameters were presented in absolute values and as pred%.

The evaluation occurred at two moments:

1. Pre-bronchodilator (pre-BD) and
2. 15 minutes post-bronchodilator (post-BD).

During the IOS evaluation, each individual underwent three measurements of 20 seconds of breathing in tidal volume. The absolute difference in the R5 parameter between two measurements for the same individual was never larger than 10%. For everyone, the measurement with the lowest R5 value was selected for statistical analysis. Regarding the spirometry evaluation, three acceptable and two reproducible maneuvers were recorded.^9^

The participants suspended the use of BDs 12 hours before the evaluation. During the assessment, all individuals used metered-dose inhalers with plastic spacer devices to inhale 400 μg of salbutamol. This evaluation was repeated at least 15 minutes after BD inhalation.

A BDR was determined according to the ATS/ERS recommendations.^9,10^ For the IOS, a BDR is characterized by a reduction of 40% in R5 or an increase of 50% in X5.^9^ In the spirometry evaluation, a BDR occurs when the differences between pre-BD and post-BD measurements of FEV_1_ and FVC are larger than 10% of the predicted values, according to ATS/ERS formula.^10^

The data was analyzed through Statistical Package for Social Sciences (SPSS) version 20.0^©^ IBM software. The Kolmogorov-Smirnov test was applied to verify data distribution. The paired Student´s t-test and the Wilcoxon Signed Rank Test were used to compare data before and after BD. The significance level of all tests was fixed at 5%. A Student's t-test comparing R5% before and after BD inhalation was used to calculate the effect size. The test showed an effect size of 0.52 in a sample of 54 individuals. We then used this value in the G*Power software to compute the power of the study and the result was 96%.

Patients were categorized into two groups: individuals with FEV_1_ at least 80% of the predicted value (G1); and individuals with FEV_1_ below 80% of the predicted value (G2). The independent Student's t-test and Mann-Whitney U test were used to compare the Δpre-post values between the groups.

## RESULTS

Eighty-three children and adolescents with CF, followed up at the referral center, were in the age group to undergo pulmonary function assessment and all were invited to this study. However, only 57 consented to participate and were assessed, but three individuals were excluded due to acute pulmonary exacerbations. Therefore, the study included 54 children and adolescents. Observing the baseline values (pre-BD), we noted that 53.8% of patients had FEV_1_ lower than 80% of the predicted value, while 74.1% had X5 above 150% of the predicted value. Their characteristics are shown in Table 1.

**Table 1:** Characterization of the studied sample.

Characteristics		Total sample (n=54)
Age (years)		9.7±2.8
Female n (%)		26 (48.1)
BMI (kg/m^2^)		16.2±2.3
BMI z-score		-0.6±1.2
Overweight n (%)		5 (9.3)
Eutrophic n (%)		45 (83.3)
Thinness n (%)		4 (7.4)
SDS classification n (%)		
	Excellent	31 (57.4)
	Good	13 (24.1)
	Average	7 (13.0)
	Poor	3 (5.6)
Variant classification n (%)		
	F508del homozygous	16 (29.6)
	F508del heterozygous	26 (48.1)
	Others	12 (22.2)
Colonization n (%)		41 (75.9)

Data are presented as mean±standard deviation or as frequency (%). BMI: body mass index; SDS: Schwachman-Doerschuk Score; F508del: cystic fibrosis´ variant.

**Figure 1:**
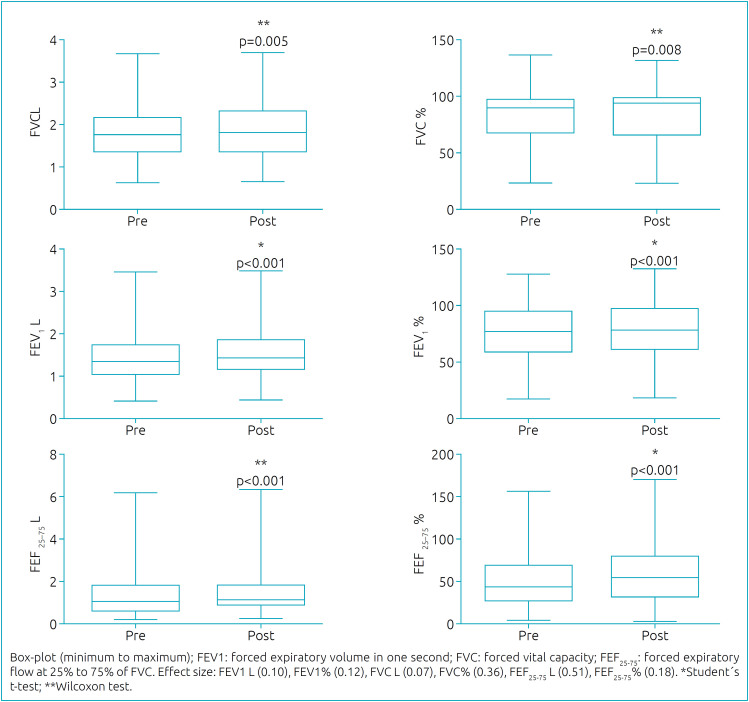
Comparison of spirometric parameters between pre- and post-BD.

**Figure 2:**
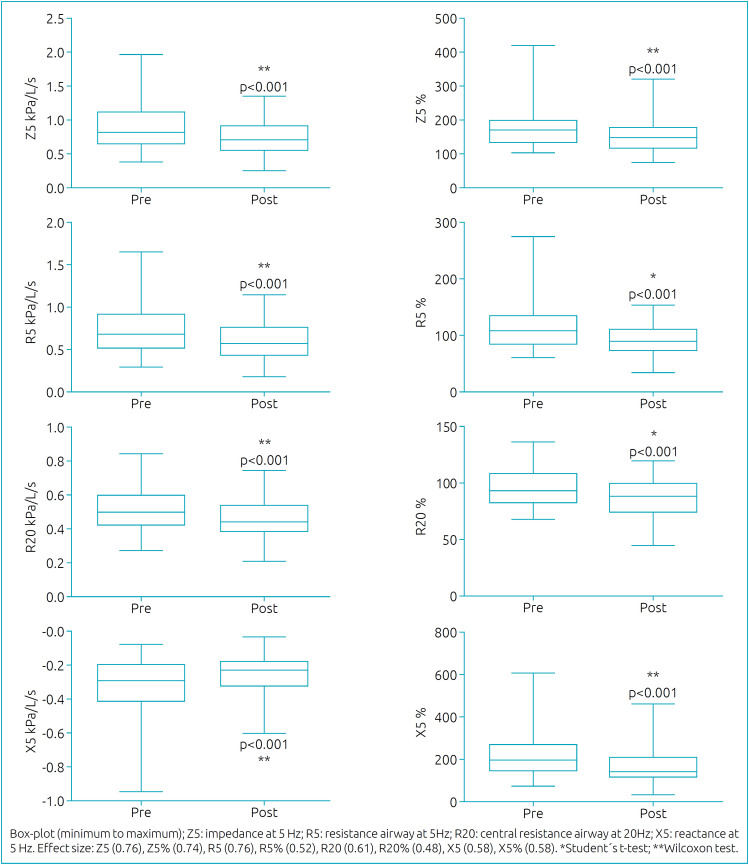
Comparison of oscillometric parameters between pre- and post-BD.

Figures 1 and 2 present the spirometric and oscillometric analyses. All the parameters showed statistical differences between pre- and post-BD, indicating improvement after BD inhalation. However, according to the ATS/ERS^10^ recommendations, only six individuals (11.1%) presented a positive BDR in FVC and FEV_1_ parameters. FEF_25-75_ was the spirometric parameter that had the greatest difference. Table 2 presents the obtained differences between pre- and post-BD assessment.

**Table 2:** Difference between pre- and post-bronchodilator (Δpre-post).

	Δpre-post		Δpre-post
FVC (L)	0.05 (0.01 to 0.08)	Z5	-0.14 (-0.19 to -0.09)
FVC%	2.07 (0.55 to 3.60)	Z5%	-31.99 (-42.83 to -21.15)
FEV_1_ (L)	0.06 (0.03 to 0.09)	R5	-0.13 (-0.16 to -0.09)
FEV_1_%	3.21 (1.54 to 4.88)	R5%	-19.95 (-26.19 to -13.71)
FEF_25-75_ (L/s)	0.13 (0.06 to 0.21)	R20	-0.04 (-0.06 to -0.03)
FEF_25-75_%	6.44 (3.02 to 9.85)	R20%	-8.27 (-11.54 to -5.00)
---	---	X5	0.07 (0.03 to 0.10)
---	---	X5%	-46.17 (-68.33 to -24.01)

Mean (95%CI): mean (95% confidence interval); Δpre-post: difference between pre-and pre-bronchodilator evaluation; FVC: forced vital capacity; FEV1: forced expiratory volume in one second; FEF_25-75_: forced expiratory flow at 25% to 75% of FVC; Z5: impedance at 5 Hz; R5: total resistance airway at 5Hz; R20: central resistance airway at 20Hz; X5: reactance at 5 Hz.

Regarding the oscillometric parameters, Z5 and R5 had the greatest post-BD improvements. In addition, X5 had a difference of about 46 points in pred%. Nonetheless, by the ERS^9^ recommendation, only three individuals (5.5%) showed a positive BDR in R5 and five individuals (9.2%) showed a positive BDR in X5. Out of the 54 individuals, 25 belonged to G1 (FEV_1_≥80%) and 29 to G2 (FEV_1_<80%). Comparing the two groups, there was no statistically significant difference.

Concerning the use of BDs and/or steroid medications: three individuals used formoterol fumarate with budesonide; three used salbutamol; one used salmeterol with fluticasone; one used an unspecified inhaled BD; nine used nasal budesonide, and two used fluticasone.

## DISCUSSION

The individuals in our study had a high SDS, meaning that they had a mild disease severity. Still, most pre-BD had FEV_1_< lower than 80% of the predicted value and X5 higher than 150% of the predicted value, indicating impairment of the respiratory system at baseline. Also, more than 70% of the individuals were colonized by some pathogen.

Regarding the BD effect, the current study showed a statistically significant improvement in spirometric and oscillometric parameters after BD inhalation. However, in both evaluations, only a few individuals achieved a positive BDR according to the ATS/ERS recommendations.^9,10^ Typically, the BDR evaluation occurs through a forced expiratory maneuver in spirometry. Although the BDR evaluation is widely used for individuals with asthma, the ATS/ERS's most recent document states that more evidence is needed regarding its cut-off points for children and young adults.^10^ Moreover, the current criteria for a positive BDR do not seem sensitive in detecting different types of COPD.^10^ Such an observation was also made by Muramatu et al., where only a few (7.7%) of the 312 spirometric data of children and adolescents with CF presented a BDR.^18^ Additionally, the authors observed improvement in the predicted percentage of FVC, FEV_1_, and FEF_25-75_.^18^

The BDR observed in around 10% of our pwCF sample is similar to the prevalence of asthma in the Brazilian population.^19^ Therefore, those individuals with CF presenting a positive BDR may be investigated for the most frequent asthma phenotypes (Th2), which include allergic, eosinophilic, and mixed asthma.^20^ In this study, even though most individuals did not show a BDR, the spirometric parameters improved after BD inhalation. Among all the spirometry parameters, FEF_25-75_ showed the greatest difference between pre- and post-BD inhalation (about 0.13 liters), suggesting an improvement in the airflow of peripheral airways. However, FEF_25-75_ is often not considered in the BDR evaluation, given that the ATS/ERS recommends analyzing only FVC and FEV_1_.^10^

Besides spirometry, the IOS has also been used to evaluate BDR.^21,22^ Although a few studies determined cut-off points for BDR through oscillation assessment — with ERS affirming that more researches with a healthy population are necessary to provide better cut-off points^9^ — the IOS complements spirometry and has been studied in individuals with asthma.^21,22^ Still, a small number of studies consider the use of IOS for BDR evaluation in people with CF. For instance, Hellinckx et al. evaluated the spirometric and oscillometric parameters of 20 individuals with CF aged 6.5 to 17.5 years.^23^ No statistical differences were found in FEV_1_ before and after BD inhalation; however, there was an improvement in resistance R6 (6Hz) and reactance X6 (6Hz) after BD, corroborating data from our study. It should be noted that the study by Hellinckx et al.^23^ was conducted before the ERS^9^ recommendation; therefore, they have not determined how many individuals presented a positive BDR in the oscillometric parameters.

Recently, Ozturk et al. used IOS to evaluate the respiratory mechanics of people with CF.^24^ In one of their analyses, BDR was evaluated in 33 children with CF (146.7±[standard deviation] 52.5 months of age) through IOS and spirometry before and 15 minutes after the inhalation of 200 μg bronchodilator medication.^24^ The authors used the older ATS/ERS reference from 2005^25^ to determine a positive BDR, and only five children had the required improvement of at least 12% in FEV_1_. In accordance with our findings, Ozturk et al. reported an improvement in all IOS parameters after BD inhalation. However, similarly to the previously mentioned study of Hellinckx et al., the authors have not used cut-off points to determine whether a child had positive or negative BDR in the IOS evaluation.

Among all the different IOS parameters evaluated in this study, Z5 and R5 had larger differences between pre- and post-BD inhalation — about 0.14 and 0.13 kPa/L/s, respectively. Since Z5 refers to the sum of all forces opposing the waves emitted by IOS, and R5 corresponds to the total airway resistance, the results observed in the present study suggest that there was a global change in the respiratory mechanics, implying an improvement in the peripheral airway.^26^ Despite the results not achieving the ERS recommendation,^9^ this improvement caused by salbutamol may contribute to the clinical condition of pwCF, assisting during physical exercise and in the penetration of other inhaled medicines such as mucolytic agents and inhaled antibiotics.

As a limitation of the present study, it is worth mentioning that the number of individuals with asthma symptoms was not controlled by a questionnaire. Furthermore, the disease severity was established with the SDS,^14^ a score that was elaborated in the 1960s, and that perhaps, due to the recent treatment advances, does not accurately represent the CF severity anymore.

Although the BDR is frequently used as a criterion for asthma diagnosis, our study shows that its response may not be as expressive in people with CF. Further studies could investigate whether BD shows a clinically meaningful effect on pwCF, in other words, if they report positive or negative changes in their clinical condition after BD inhalation. This information could support the prescription of BD, once most referral centers worldwide routinely recommend BD and inhaled corticosteroids to pwCF.^5^ The ideal situation would be to individually verify if the patients inform changes in their daily living after BD inhalation and present positive BDR, which could mean a CF-asthma overlap syndrome.^27^

In conclusion, the individuals assessed in this study did not meet the BDR classification criteria. However, the results of our study showed that the use of BD in children and adolescents with CF might lead to improvements in pulmonary volumes as well as airway resistance, which corroborates our primary hypothesis.

## Data Availability

The database that originated the article is available with the corresponding author.
